# Endoloop ligation-assisted resection of bleeding ulcerated Brunner’s gland adenoma

**DOI:** 10.1055/a-2366-5234

**Published:** 2024-08-13

**Authors:** Arthur Jourdain, Antoine Guilloux, Jeanne Salesse, Marie Lequoy, Romain Leenhardt, Xavier Dray, Marine Camus Duboc

**Affiliations:** 1 37117 Sorbonne University, Center for Digestive Endoscopy, Hospital Saint-Antoine, APHP, Paris, France; 2 37117 Sorbonne University, Hepatology, Hospital Saint-Antoine, APHP, Paris, France; 3 37117 Anatomopathology, Hospital Saint-Antoine, Paris, France

Brunner’s gland adenoma is a rare benign polyp located in the proximal duodenum above the duodenal papilla 1 . It arises due to the proliferation of Brunner’s glands and can result in polyps larger than 2 cm. These large lesions may lead to complications such as duodenal obstruction, bleeding, and duodenal intussusception. Bleeding primarily occurs due to traumatic ulceration of the polyp, caused by the back and forth movements of the polyp’s head against the opposite duodenal wall. Endoscopic resection is the preferred treatment for Brunner’s gland adenoma and can be performed using polypectomy, endoscopic mucosal resection, or endoscopic submucosal dissection 2 .

We report the case of an 88-year-old patient on rivaroxaban who presented with melena, acute anemia, and a hemoglobin level of 8.2 g/dL at admission. He required a transfusion of three red blood cell units during hospitalization. Gastroscopy revealed a large pedunculated polyp located on the anterior wall of the duodenal bulb, with ulceration at its apex ( Fig. 1 , Fig. 2 , Fig. 3 ). No other source of bleeding was identified.

**Fig. 1:**
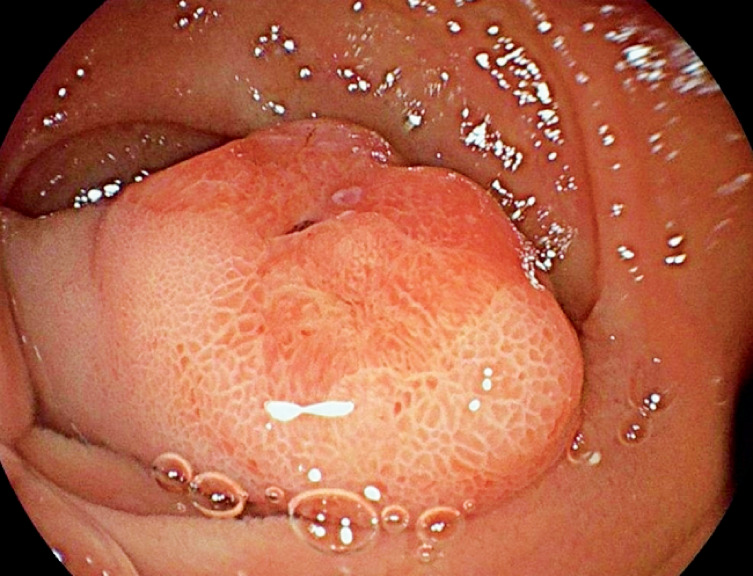
Gastroscopic image of the head of Brunner’s gland adenoma in an 88-year-old patient with melena.

**Fig. 2:**
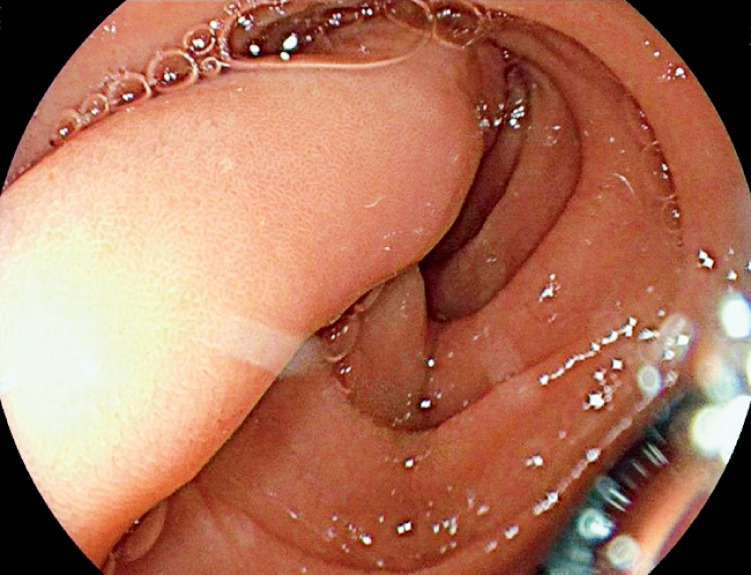
Gastroscopic image of the stalk of Brunner’s gland adenoma in an 88-year-old patient with melena.

**Fig. 3:**
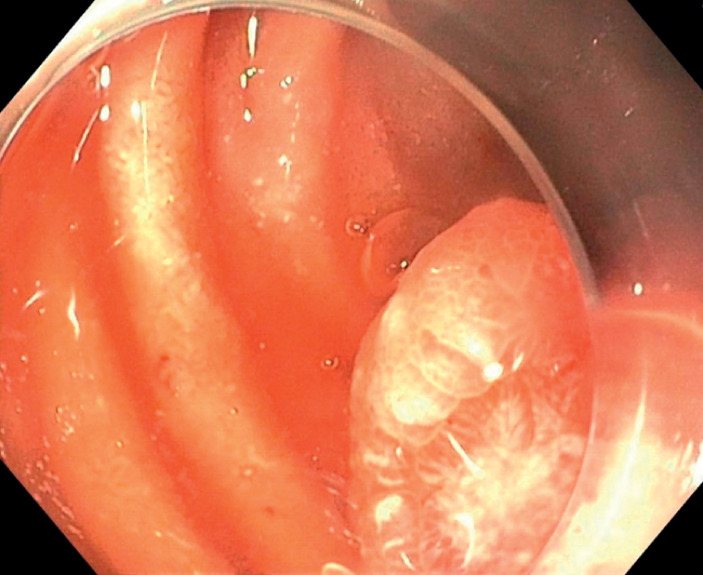
Gastroscopic image of ulceration on the head of a polyp in an 88-year-old patient with melena.

We decided to perform endoloop ligation-assisted resection to facilitate the polypectomy and prevent post-resection bleeding. After deploying, tightening, and releasing the Endoloop system (Olympus PolyLoop Ligation Device, Tokyo, Japan) ( Fig. 4 ), a hot polypectomy snare (Fujifilm Medwork, Höchstadt/Aisch, Germany) was used to safely excise the polyp without any bleeding ( Video 1 ).

**Fig. 4:**
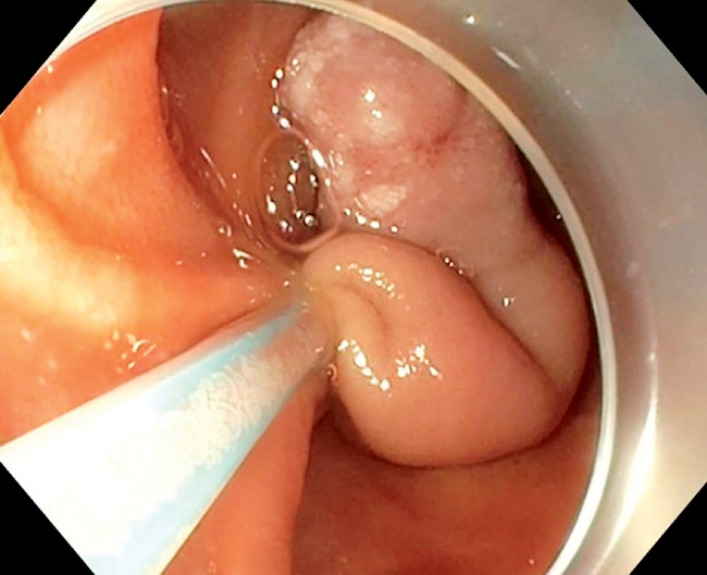
Placement of an endoloop system for endoloop ligation-assisted resection of a polyp.

Endoloop ligation-assisted resection of a bleeding ulcerated Brunner’s gland adenoma. This safe and effective procedure prevents post-resection bleeding.Video 1

Histological analysis revealed ulcerated intestinal mucosa with abnormally enlarged Brunner’s glands in the submucosa ( Fig. 5 ), leading to the diagnosis of an ulcerated Brunner’s gland adenoma.

**Fig. 5:**
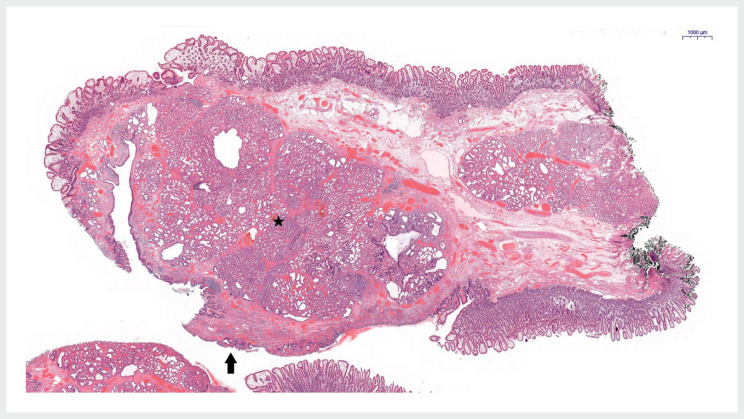
Microscopic features of a Brunner’s gland adenoma (star) with a lobular architecture and ulceration (arrow) with normal duodenal mucosa on both sides.

Brunner’s gland adenoma typically does not require resection as it is a benign condition 3 . However, in cases of large and symptomatic polyps, endoscopic treatment is appropriate. Endoloop ligation-assisted resection of Brunner’s gland adenoma is a safe and effective method for preventing post-procedure bleeding.

Endoscopy_UCTN_Code_TTT_1AO_2AG_3AB

## Notes

Endoscopy E-Videos https://eref.thieme.de/e-videos
*E-Videos* is an open access online section of the journal *Endoscopy* , reporting on interesting cases and new techniques in gastroenterological endoscopy. All papers include a high-quality video and are published with a Creative Commons CC-BY license. Endoscopy E-Videos qualify for HINARI discounts and waivers and eligibility is automatically checked during the submission process. We grant 100% waivers to articles whose corresponding authors are based in Group A countries and 50% waivers to those who are based in Group B countries as classified by Research4Life (see: https://www.research4life.org/access/eligibility/ ). This section has its own submission website at https://mc.manuscriptcentral.com/e-videos .
